# Tumor-derived exosomes as promising tools for cancer diagnosis and therapy

**DOI:** 10.3389/fphar.2025.1596217

**Published:** 2025-05-15

**Authors:** Xirui Wang, Yanfang Liu, Yaowen Jiang, Qinghua Li

**Affiliations:** ^1^ Department of Biomedical Engineering, School of Medical Imaging Xuzhou Medical University, Xuzhou, China; ^2^ Department of Central Laboratory, Affiliated People’s Hospital of Jiangsu University, Zhenjiang, China; ^3^ Institute of Medical Imaging and Artificial Intelligence, Jiangsu University, Zhenjiang, China

**Keywords:** tumor-derived exosomes, cancer diagnosis, cancer vaccine, cancer therapy, drug delivery, engineered exosomes

## Abstract

Mounting evidences indicated that cancer cell-derived exosomes (TDEs) contribute to cancer progression and metastasis by reshaping the tumor microenvironment (TME) and interfering immunity response. TDEs contain unique biomolecular cargo, consisting of protein, nucleic acid, and lipids. In recent years, TDEs have been used as potential disease therapeutics and diagnosis biomarkers and prime candidates as delivery tools for cancer treatment. In the present review, we firstly summarized TDEs biogenesis and characteristic. Also, the role of TDEs in cancer cell metastasis and invasiveness, drug resistance, and immunosuppression was mentioned via cell-cell communication. Additionally, we concluded the current strategies for TDE-based cancer therapy, including TDEs inhibition and clearance, usage as therapeutic drug delivery vector and cancer vaccines. Furthermore, combination therapy with engineered TDEs were summarized, such as radiotherapy, photodynamic therapy, photothermal therapy, and sonodynamic therapy. Consequently, the above opens up novel and interesting opportunities for cancer diagnosis and prognosis based on TDEs, which is prospective to accelerate the clinical translation of TDEs for cancer therapy.

## 1 Introduction

Exosomes are extracellular nanoscale vesicles found ubiquitously in blood, urine, saliva, cerebrospinal fluid, pleural fluid, and breast milk, typically ranging in size of 40–150 nm secreted by living cells (Kalluri, 2016; Kalluri and McAndrews, 2023). They contain various active messenger materials, including lipids, proteins, nucleic acid (such as messenger ribonucleic acid (mRNA), long non-coding RNA (lncRNA), microRNA, deoxyribonucleic acid (DNA)), and so on (Yokoi et al., 2019; Yu et al., 2023). Exosomes are considered to be the key mediators of intercellular transportation, which vary depending on the origin, the physiological or pathological conditions of the cell (Li and Nabet, 2019). Tumor cell-derived exosomes (TDEs) attract the most interest because of their roles in cancer development and progression depending on the intercellular communication (Wee et al., 2019; Hyung et al., 2023; Li et al., 2024). Due to the ability of retaining the original characteristics of derived cancer cells, the ubiquitously TDE can both regulating the neighboring cancer cell and shaping the surrounding microenvironment by communicating with multiple kinds of cells, such as immune cells, cancer-associated fibroblasts (CAFs), to promote tumor growth (Luo et al., 2023; Tsunedomi et al., 2023). Moreover, the immunosuppressive microenvironments creation by inhibiting the activity of immune cells, such as T-cells and natural killer (NK) cells, and secreting immunosuppressive cytokines to silence immune responses (Chen G. et al., 2018; Hosseini et al., 2022; Vulpis et al., 2022).

Understanding the role of TDEs in tumor progression and metastasis has accelerated the development of cancer therapeutic strategies. Selectively clearance of the TDEs can potentially achieve therapeutic benefits by hampering the essential cell-cell communication required for cancer progression (Tu et al., 2022; Shin et al., 2023). In addition, with to the advantage of biocompatibility, bioavailability, immunogenicity, better stability, cellular uptake mechanism, and capability of higher payloads, TDEs exhibit significant promise in overcoming the limitations of conventional synthetic nanoparticle (such as liposomes, polymeric nanoparticles, and inorganic nanoparticles) and virus-based techniques in targeted therapy for delivery vehicles of therapeutic agents or cancer vaccines (Wolfers et al., 2001; Qiao et al., 2020; Huang L. et al., 2022; Chu et al., 2025). As the TDEs carry similar surface proteins from their cells of origin, they display potential ability to cancer diagnosis, risk stratification, monitoring, and treatment decision-making based on their surface markers. This review provides a comprehensive overview of the current understanding of TDEs biogenesis as well as its biological effect. TDEs-based liquid biopsies for tumor biomarkers and clinical implementation in the field of cancer diagnosis are also described. Subsequently, this review emphasizes on therapy strategies based on TDEs. Finally, existing challenges, as well as the future direction for TDEs-targeting cancer therapy, are discussed.

## 2 TDEs biogenesis and characteristic

**FIGURE 1:**
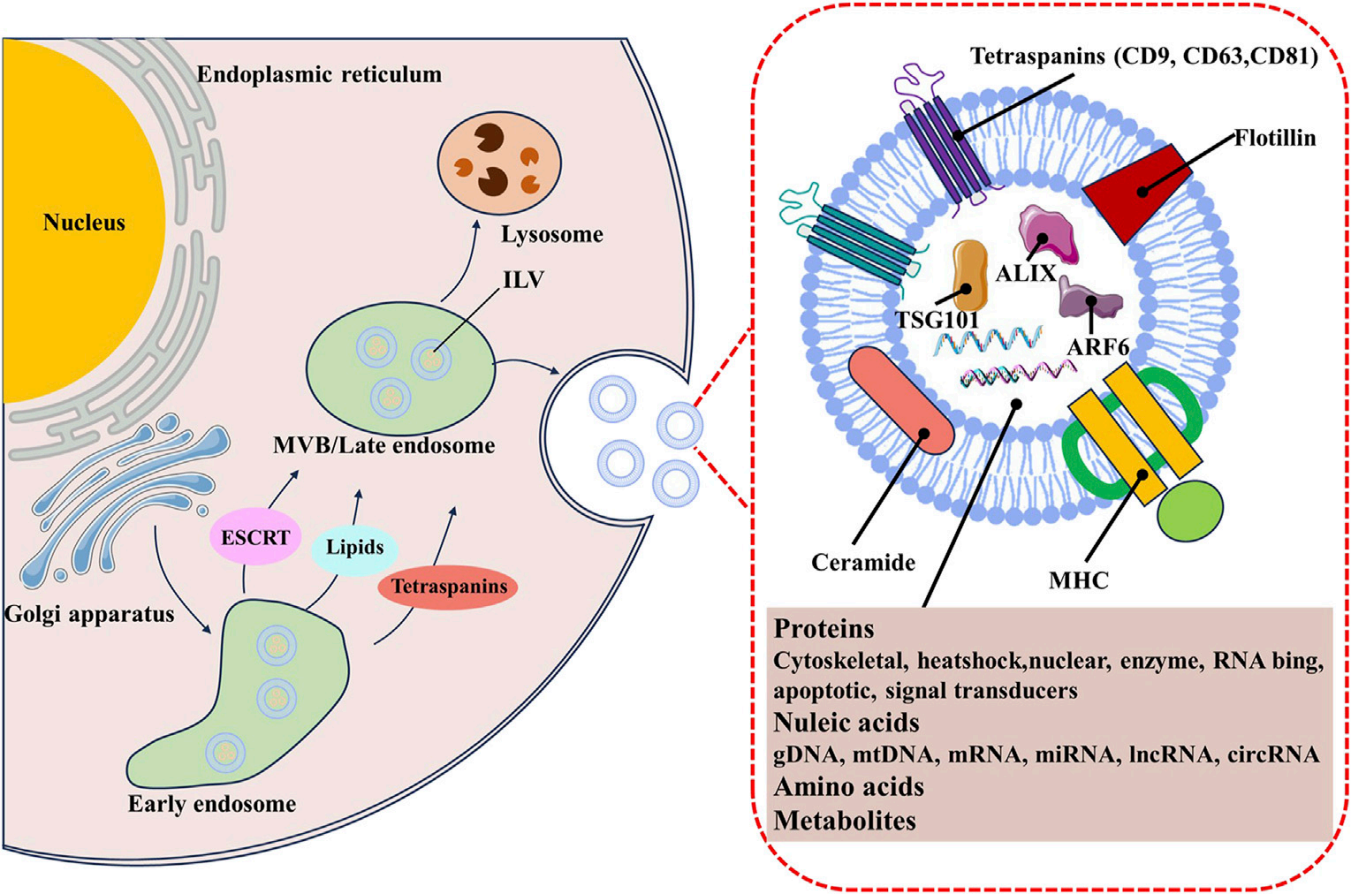
The biogenesis and contents of TDE.

The biogenesis and secretion of exosome are complex processes, involving double invagination of the plasma membrane, and subsequent fusion of multivesicular bodies (MVBs) containing intraluminal vesicles (ILVs) (Kalluri and LeBleu, 2020; Han et al., 2022). These start with the inward membrane budding of early sorting endosomes (ESEs) with the help of the trans-Golgi network (Teng and Fussenegger, 2021). The ESEs then mature into late-sorting endosomes (LSEs) and eventually generate MVBs by the inward budding of the endosomal membrane. Finally, ILVs are ultimately secreted as exosomes through MVBs fusion with the plasma membrane or lysosomes, or autophagosomes for degradation (Théry et al., 2002; Arya et al., 2024). It is well known that exosomes can be generated by endosomal sorting complexes required for transport (ESCRT)-dependent or ESCRT-independent pathways (Juan and Fürthauer, 2018; Wei et al., 2021). The ESCRT machinery primarily includes four complexes (named ESCRT-0, ESCRT-I, ESCRT-II, ESCRT-III) and some accessory components such as apoptosis-linked gene 2-interacting protein X (ALIX), syntenin, and vacuolar protein sorting 32 (VPS32) (Baietti et al., 2012; Larios et al., 2020; Salas et al., 2022). Exosomes can also be generated through ESCRT-independent pathways. Those protein similar to the lipid raft microdomains, including phospholipids, tetraspanins, ceramides, and neutral sphingomyelinases (nSMases), have been expounded to participate in exosome biogenesis through additional pathways (Trajkovic et al., 2008; Van Niel et al., 2011; Niekamp et al., 2022) (Figure 1).

As an important subset of exosomes, TDEs share the common mechanisms of biogenesis. Cancer cells usually secrete more exosomes than normal cells development owing to its intrinsic oncogenic signaling and the located special tumor microenvironment, such as hypoxia, inflammation and low pH (Taylor et al., 2020). such as hypoxia, inflammation and low pH (Meng et al., 2019; Tian et al., 2019). Lung cancer cells generate more exosomes under hypoxic conditions than the normoxic condition (Hsu et al., 2017). King et al. founded breast cancer cell-derived exosomes significantly increased in a hypoxia-inducible factor (HIF)-1α-dependent manner (King et al., 2012). HIF activated RAB22A gene lead to the enhanced secretion of TDEs (Wang et al., 2014). The p53^−/−^cancer cells under the hypoxia, inflammation, and cellular stress condition also produce more TDEs through upregulated the TDEs biogenesis-related genes, including TSAP6, Chmp4C, and nSmase2 (Bebelman et al., 2021). Extracellular acidity is also a key regulator for the TDEs biogenesis and secretion. The acidity of TME facilitates the release of TDEs in prostate cancer (Logozzi et al., 2017). It has been elucidated that acidic conditions could release more TDEs than that under buffered conditions by enhancing ganglioside monosialodihexosylganglioside 3 (GM3) content responsible for the increased fusion efficiency and caveolin-1, which is an important protein involved in melanoma progression (Parolini et al., 2009).

TDEs contain or express various bioactive biomolecules derived from their parental cells. Therefore, they have the intrinsic ability to control complex biological functions. TDEs-mediated communications between cancer cells and other types of cells increase the invasiveness of cancer cells, drug resistance and immunosuppression (Milman et al., 2019; Liu et al., 2021a; Hinzman et al., 2022). TDEs can act as messengers to regulate both cancer cells and their microenvironment, which is favorable for cancer growth and metastasis. TDEs cargos, especially oncogenic microRNAs (miRNAs), are considered the major contributor to tumor initiation and growth (Wang et al., 2019; Zhang et al., 2024). Breast cancer and pancreas cancer derived exosomes can transfer oncogenic miRNAs into non-tumourigenic pancreatic normal epithelial cells s and induce the malignant transformation (Hinzman et al., 2022). Additionally, miRNAs in non-small cell lung cancer-derived exosomes can facilitates distant tumor growth by targeting angiogenesis related genes such as vascular endothelial growth factor (VEGF) (Ma et al., 2021; Zhao et al., 2022). Proteins and miRNAs encapsulated into TDEs activate the transforming growth factor-β (TGF-β) signaling pathway to reprogram normal fibroblasts into CAFs, contributing to tumor progression and metastasis (Sung et al., 2020). Breast cancer exosome-derived miR-122 suppresses pyruvate kinase and subsequent glucose uptake in the lungs to promote metastasis (Fong et al., 2015). In addition to their roles in tumor progression, TDEs also contribute to drug resistance, including chemotherapy, immunotherapy, and radiotherapy (Cheng et al., 2024). A variety of drug efflux pumps in TDEs, such as P-glycoprotein (P-gp) mediate direct export of drugs from cancer cells TDEs deliver (Yang et al., 2022). In addition, other proteins in TDEs could inhibit apoptosis, promote survival and proliferation, divert protein splicing toward more oncogenic subtypes in recipient cancer cells (Ma et al., 2014; Ning et al., 2017). Nucleic acids present in TDEs, including mRNA, lncRNA, circRNA, and mitochondrial DNA (mtDNA), also play critical roles in mediating therapy resistance through interfering with diverse signaling pathways associated with autophagy, metabolism, and epithelial-to-mesenchymal transition (EMT) (Santos et al., 2018; Zhang et al., 2019). Moreover, acquired drug resistance could also be achieved with the help of drug-resistant cancer cells derived TDEs. In addition, TDEs may also induce immunosuppression through interacting with immune cells, such as T cell, B cells, natural killer cells, dendritic cells, and macrophages (Hao et al., 2022). TDEs can deactivate CD8^+^ T cell antitumor function through PD-1/PD-L1 binding and block dendritic cell maturation and migration in a PD-L1-dependent manner (Chen G. et al., 2018). TDEs also present suppressive effects on natural killer cells, macrophages in the TME, facilitating immune escape (Cheng et al., 2021; Hosseini et al., 2022). The liberation of TGF-β from exosomes released by tumor cells also significantly contributes to tumor biology including tumor progression, differentiation and immune evasion (Hosseini et al., 2023; Wang H. et al., 2024). Chao Ni et al. found that breast tumor cell-derived exosomes can transport lncRNA SNHG16, leading to the upregulation of CD73 expression in Vδ1 T cells. SNHG16 functions as a competing endogenous RNA (ceRNA) by sequestering miR-16-5p, leading to the downregulation of its target gene SMAD5, which ultimately activates the TGF-β1/SMAD5 pathway, contributing to immunosuppression (Ni et al., 2020). Furthermore, TDEs also displayed the unique biocompatibility, immunogenicity, stability, pharmacokinetics, biodistribution, and cellular uptake mechanism, all of which conferred them to be the promising diagnostic and target therapy both at the basic and applied levels.

## 3 TDEs in cancer diagnosis

TDEs are rich in biological fluids and released continuously by all living cancer cells, rendering them attractive as minimally invasive liquid biopsies with the potential for longitudinal sampling to follow cancer progression. TDEs biogenesis enables the capture of a complex extracellular and intracellular molecular cargo for comprehensive, multiparameter diagnostic. All these biological cargos involved in the tumorigenic process and surface molecules on TDEs facilitate their immune capture and enrichment.

Liquid biopsy mainly focused on the characterization and analysis of extracellular nucleic acids. Specific nucleic acids or groups of nucleic acids in TDEs may provide diagnostic or prognostic potential in cancer. Han Wang and his colleagues conducted a study showing that highly expressed CLDN7 exosomes promote migration and invasion in triple-negative breast cancer (TNBC) cells and serve as prognostic biomarkers for the TNBC (Wang et al., 2023). Skog et al. showed that tumor-derived mutations could be detected via mRNA in exosomes isolated from plasma of patients (Skog et al., 2008). 11,000 distinct mRNAs were detected in exosomes derived from colorectal cell line supernatant, indicating the potential diagnosis biomarker for the colorectal cancer patients (Hong et al., 2009). Oncogenic and tumor-suppressor miRNAs in TDEs differentially expressed between cancer cells and normal cells, enhancing their usefulness in early diagnosis. TDEs isolated from different cancers, including glioblastoma, lung cancer, and breast carcinoma, have distinct miRNA profiles, indicating their powerful in diagnosis the special cancer (Li et al., 2023). The amounts of DNA can be found in TDEs, which can be used to detecting cancer-associated mutations in serum exosomes (Kahlert et al., 2014; Thakur et al., 2014; Wang L. et al., 2018). Kras mutation cancer cell derived TDEs was reported to be enrich for miR-100 (Cha et al., 2015). Certain miRNA species, eg, miR-21, were found to be highly associated with glioblastomas and pancreatic, colorectal, liver, breast, ovarian, esophageal, bladder and prostate cancer, and miR-21 expression levels correlated with the disease presence, progression, and response to therapy (Salehi and Sharifi, 2018). However, whether TDEs isolated from human contain DNA remains contentious and quantitative studies are required.

Proteins located on the surface and within TDEs may also be used as cancer biomarkers. Total protein levels and the content of individual proteins in TDEs varies between cancer and normal patients, providing prognostic information. TGF-β1 were found to be significantly elevated in TDEs isolated from acute myeloid leukemia (AML) patients plasma, which can inhibit activation and functions of NK cells to downregulate cytotoxicity mediated by NK cells (Hong et al., 2014). Epidermal growth factor receptor variant III (EGFRvIII) in TDEs isolated from plasma with brain cancer have been successfully explored as the diagnosis biomarker (Choi et al., 2018). Also, human epidermal growth factor receptor 2 (HER2) as a breast cancer marker has also been widely reported (Wang M. et al., 2018; Nanou et al., 2020). Prostate-specific antigen (PSA) in plasma TDEs can distinguish prostate cancer from benign hyperplasia (Logozzi et al., 2019). PD-L1^+^ TDEs were placed as promising biomarker of prognosis and response to ICIs in melanoma and other cancers (Chen G. et al., 2018). In addition to nucleic acids and proteins, lipids and glycans in TDEs are emerging as potential biomarkers. Compared with healthy donors, lipidomic analysis of exosomes from colorectal cancer patients displayed a promising approach for diagnosing, staging, and subtyping colorectal cancer (Elmallah et al., 2022). Urinary exosomal lipids have a high diagnostic capacity for prostate cancer as non-invasive prostate cancer biomarkers (Skotland et al., 2017). Glycans and glycome also display as the potential powerful tools to diagnose prostate cancer, and to accurately determine tumor aggressiveness and patient prognosis (Scott and Munkley, 2019) (Table 1).

**TABLE 1:** Diagnosis biomarkers from TDEs.

Biomarker type	Cancer type	Biomarker	References
mRNA	ccRCC	CUL9/KMT2D/PBRM1/PREX2/SETD2	He et al. (2022)
	Prostate cancer	CDC42/IL32/MAX/NCF2/PDGFA/SRSF2	Ji et al. (2021)
	Glioblastoma	MGMT/APNG	Shao et al. (2015)
miRNA	NSCLC	let-7/miR-21/miR-24/miR-486	Jin et al. (2017)
		Adenocarcinoma: miR-181-5p/miR-30a-3p/miR-30e-3p/miR-361-5p	
		SCC: miR-10b-5p/miR-15b-5p/miR-320	
	NSCLC	miR-21/TTF-1 mRNA	Yang et al. (2018)
lncRNA	Prostate cancer	SAP30L-AS1/SChLAP1	Wang et al. (2018c)
	GBM	HOTAIR	Tan et al. (2018)
DNA	Pancreatic cancer	KRAS/p53 mutations	Kahlert et al. (2014)
	PCCs/PGLs	RET/HIF2A/VHL/SDHB	Wang et al. (2018a)
Protein	Pancreatic cancer	GPC1/EphA2	Yu et al. (2023)
	Brain cancer	EGFRvIII	Choi et al. (2018)
	Breast cancer	HER2	Nanou et al. (2020)
	NSCLC	AHSG/ECM1/carcinoembryonic antigen	Niu et al. (2019)
Lipids	Prostate cancer	Cholesterol	Skotland et al. (2017)
	Breast cancer	Phosphatidylserine	Sharma et al. (2017)
Glycoconjugates	NSCLC	MUC1	Pan et al. (2019)
	Breast Cancer	glycoproteins	Chen et al. (2018b)

Abbreviations: NSCLC, non-small cell lung cancer; GBM, glioblastoma multiforme; ccRCC, clear cell renal cell carcinoma; MGMT, O6-methylguanine DNA, methyltransferase; APNG, alkylpurine-DNA-N-glycosylase; PCCs, pheochromocytomas; PGLs, paragangliomas; GPC1, glypican-1; EphA2, ephrin type-A, receptor 2; AHSG, alpha-2-HS-glycoprotein; ECM1, extracellular matrix protein 1.

## 4 Treatment strategies in anticancer therapy targeting TDEs

As TDEs play an essential role in facilitating cancer growth and metastasis, emerging strategies are employed for targeted TDEs by inhibiting their biogenesis and secretion, disrupting their systemic circulation, and reducing their uptake by recipient cells (Li et al., 2022). In addition, TDEs could serve as the therapeutic agents vehicles for targeted delivery of drug payload(s) (Zheng et al., 2018; Qiao et al., 2020). In contrast to synthetic nanoparticles, such as liposomes, biomimetic TDEs are efficient at entering other cells and can deliver a functional cargo with minimal immune clearance upon exogenous administration in mice. Furthermore, cancer-specific antigens in TDEs make them natural vaccines for boosting the immunogenicity of personalized anticancer therapy (Naseri et al., 2020). Therefore, TDEs-based treatment strategies may be promising for the precision cancer therapy, which is prospective to accelerate the clinical application for cancer-targeted therapy.

### 4.1 Clearance strategies for TDEs

**FIGURE 2:**
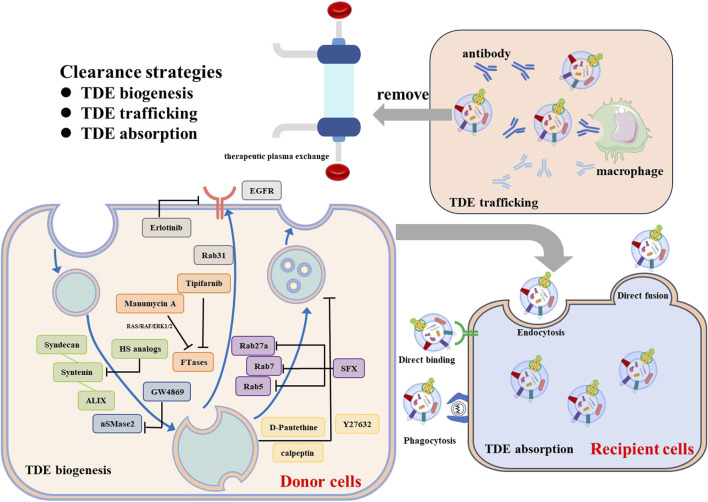
Clearance strategies for TDEs.

The biogenesis of TDEs is multiple mechanisms. Inhibiting TDEs could be achieved by interrupting the key processes involved in EV biogenesis with pharmacological inhibitors or genetic manipulation (Figure 2). ESCRT machinery is typically exploited to generate TDEs, interfering with ESCRT-related signaling pathways may reduce the TDEs production. Im et al. revealed that sulfisoxazole (SFX), an oral antibiotic, selectively inhibited the secretion of TDEs from breast cancer cells by suppressing the transcription of Rab GTPases (Rab5, Rab7, and Rab27a) and ESCRT components (ALIX, VPS4B) (Im et al., 2019). Colombo et al. found that the silencing of HRS, STAM1, or TSG101 led to a reduction in TDEs secretion and exosomal major histocompatibility complex (MHC) class II (Colombo et al., 2013). The syndecan-syntenin-ALIX signaling showed promising outcomes in inhibiting the TDEs biogenesis of cancer. Heparan sulfate (HS) analogs specifically and efficiently inhibited TDEs secretion by targeting syndecan-syntenin- ALIX, leading to weakened tumor proliferation and invasion (Wu et al., 2021). The disruption of syndecan-syntenin-ALIX by RNAi lead to the reduced secretion of exosomes in MCF-7 human breast cancer cells (Baietti et al., 2012). ESCRT-independent ways also paly important role in biogenesis TDEs. nSMases-ceramide pathway, the most studied ESCRT-independent pathway, controlled MVBs budding from the plasma membrane. Knockdown of nSMase2 in cancer cells resulted in decreased TDEs secretion and suppressed metastasis (Poggio et al., 2019). GW4869, the most commonly used sphingomyelinase inhibitor, inhibited the secretion of exosomes from a variety of tumor cells, including breast cancer cells, bladder cancer cells, epidermal cancer cells and malignant melanoma cells (Rezaie et al., 2022). Rab31 regulated exosome biogenesis in HeLa cells through an ESCRT-independent pathway and drove the sorting of protein cargos such as EGFR (Wei et al., 2021). Sasabe et al. employed erlotinib, an EGFR inhibitor, to suppress the TDEs secretion in oral squamous cell carcinoma (OSCC) (Sasabe et al., 2022). The genomic mutations that contribute to the aberrant biogenesis and secretion of TDEs could provide specific therapeutic targets. Datta et al. found manumycin-A (MA), which selectively affected RAS/RAF/ERK1/2 by targeting farnesyltransferases (FTases), displayed high efficacy in inhibiting TDEs secretion in castration-resistant prostate cancer (CRPC) cells (Datta et al., 2017). Tipifarnib, a FTase inhibitor, was also found to inhibit TDEs release from the prostate cancer cells, suggesting that FTase inhibitors can function as a class of potential effectors to block TDEs (Datta et al., 2018). Moreover, interfering with the lipid composition and blocking cytoskeleton reorganization also leads to a decreased level of MVB biogenesis and inhabitation of TDEs secretion. D-Pantethine, a cholesterol synthesis inhibitor, reduced the biogenesis of MVB both *in vitro* and *in vivo* (Roseblade et al., 2015). Calpain inhibitors, calpeptin, and Rho-associated protein kinases (ROCK), Y27632, can block MVB formation and release by targeting cytoskeletal proteins (Li et al., 2012).

Disrupting TDE’s cell-cell communication is another strategy for treating cancer. Phagocytotic clearance of immune cells, especially macrophages, or competitive binding of aptamer-ligand within the circulatory system can effectively reduce the uptake of TDEs. Nishida Aoki et al. found that injecting antibodies against CD9 and CD63 can reduce cancer metastasis development by increasing the cellular uptake of TDEs by macrophages (Nishida-Aoki et al., 2017). Kimura et al. discovered that using anti-cytoskeleton-associated protein 4 (CKAP4) monoclonal antibodies (mAbs), a specific biomarker for pancreatic ductal adenocarcinoma (PDAC)-derived exosomes, can suppress tumor growth *in vivo* (Kimura et al., 2019).

In addition to the antibodies, the removal of TDEs from the circulation through therapeutic plasma exchange (TPE) is another promising approach (Orme et al., 2020). The plasma from patients was extracted by apheresis equipment and replaced by colloid solutions to discard the circulating exosomes. However, due to the disadvantages with high cost and high invasiveness significantly limited its clinical translation. Moreover, interfering with exosome-driven signaling in recipient cells can potentially relieve exosome-mediated intercellular communication and further inhabit exosome mediated tumor promoting effect. Selective inhibition of PI3K signaling by wortmannin and LY294002 reduced the TDEs uptake by macrophages in a dose-dependent manner (Fitzner et al., 2011). Cytochalasin D and Latrunculin A, actin polymerization inhibitors, required for cytoskeletal remodeling, blocked the TDEs adsorption by human umbilical vein endothelial cells (HUVECs) (Svensson et al., 2013). Despite significant efforts to impede the transport of TDEs, achieving specificity remains a challenge due to the heterogeneity of these vesicles. More efforts to target and eliminate patients’ specific TDEs biomarkers are still needed.

### 4.2 TDEs as delivery tools for cancer treatment

As the naturally secreted nanoparticles, TDEs are good delivery vehicles for anticancer therapy. Compare to synthetic nanoparticles, TDEs exhibit lower toxicity, immunogenicity, higher stability, and targeting ability, extended drug retention and minimized off-target effects (Nam et al., 2020). TDEs loaded with various chemotherapy drugs or bioactive molecules have been widely used in many anti-cancer strategies. Various chemotherapeutics, including doxorubicin, paclitaxel, cisplatin, and methotrexate, have been encapsulated into TDEs to realize the target delivery. Qiao et al. reported that enveloped doxorubicin inside TDEs could increase drug retention in solid tumors and improved the antitumor efficacy (Qiao et al., 2020). Akhil et al. developed doxorubicin-conjugated gold TDEs nanoparticles displayed preferential cytotoxicity on cancer cells and minimal activity against normal cells (Srivastava et al., 2016). Similarly, paclitaxel and cucurbitacin B co-encapsulated into micelles decorated with TDEs membranes selectively target to homotypic circulating tumor cells and penetrate deeper tumor, suppressing the tumor growth and metastasis (Wang K. et al., 2020). Further, engineered exosomes are made their way into history for their tumor-targeting competence and reduced system toxicity. Paclitaxel-loaded engineered TDEs not only inhibit tumor growth, but also prevent recurrence and metastasis in breast cancer-bearing mice (Wang K. et al., 2020). Apart from the superior cancer-targeting ability, TDEs can help to overcome the drug-resistance. Intrathoracic administration of cisplatin-packaging TDEs derived from A549 human lung cancer cell lines effectively eradicated cisplatin-resistant cancer cells (Ma et al., 2016).

Apart from conventional small molecular drugs, TDEs are the most efficient nucleic acids (such as miRNA, siRNA, DNA, etc.) transporter tools and are used for therapeutic gene delivery (JC Bose et al., 2018). miRNA-126 loaded breast cancer (MDA-MB-231) cell-derived exosomes strongly suppressed A549 lung cancer cell proliferation and migration through the interruption of the PTEN/PI3K/AKT signaling pathway (Nie et al., 2020). Similarly, engineered 4T1-derived exosomes loaded with therapeutic anti-miRNAs and low dose doxorubicin synergistically inhibit of tumor growth, reduce of lung metastases, and extend the survival of 4T1 tumor-bearing mice (Bose et al., 2022).The novel hybrid NPs, consisting of exosomes derived from MCF7 cells and functionalized poly (amidoamine) (PAMAM) dendrimers was used as a gene delivery vehicle to enhanced siRNA delivery (over 2-fold) to cancer cells, compared to dendrimers alone (Nair et al., 2023). The advent of the novel gene-editing method CRISPR-Cas9 has considerably altered in the field of gene therapy. Kim et al. reported that exosomes derived from ovarian cancer cells engineered to carry CRISPR/Cas9 targeting poly (ADP-ribose) polymerase-1 (PARP-1) could target ovarian tumors more effectively than those derived from normal epithelial cells, thereby significantly reducing tumor growth (Kim et al., 2017). Moreover, numerous studies have attempted to utilize membrane proteins as therapeutic agents due to their beneficial attributes. However, structure complexity and difficulty producing their hydrophobic regions can cause technical difficulties and significantly hinder its clinical translation. Currently, most protein-delivering exosomes are derived from normal cells, mainly HEK293T (Koh et al., 2017; Cho et al., 2018). Yoshinobu developed a genetically engineered TDEs modified with a fusion protein SAV−LA and a pH-sensitive fusogenic peptide GALA to enhance tumor antigen presentation capacity *in vitro* (Morishita et al., 2017). However, more TDE-based protein-delivering systems are deficient and need more research to elucidate (Table 2).

**TABLE 2:** Summary of TDE-based delivery systems for the treatment of cancer.

Drug type	Cancer type	TDE source	Cargo	References
Chemotherapy drugs	Fibrosarcoma/Cervical cancer	HT1080/HeLa cells	Doxil	Qiao et al. (2020)
	Lung cancer	H1299/YRC9	doxorubicin	Srivastava et al. (2016)
	Lewis lung carcinoma	A549	Cisplatin	Ma et al. (2016)
	Lung cancer/breast cancer/lung metastasis	H22/Bel7402/B16F10	Doxorubicin	Yong et al. (2019)
	Breast cancer	MDA-MB-231	Paclitaxel/cucurbitacin B	Wang et al. (2020b)
	Prostate cancer	LNCaP/PC-3	Paclitaxel	Saari et al. (2015)
	Brain tumor	Tumor cells (such as GL26-Luc/BV2)	JSI124 (cucurbitacin I)	Zhuang et al. (2011)
	Glioblastoma	U-87 cell	Paclitaxel	Salarpour et al. (2019)
	Pancreatic cancer	PANC-1/MIA PaCa-2	Curcumin	Osterman et al. (2015)
	Osteosarcoma	143B/MG63	Methotrexate	Wang et al. (2024b)
Nucleic acid	Breast cancer	4T1/HepG2/SKBR3	anti-miR-21	JC Bose et al. (2018)
	Lung cancer	MDA-MB-231	miRNA-126	Nie et al. (2020)
	Breast cancer/lung metastasis	4T1	antimiRNA-21/antimiRNA-10b	Bose et al. (2022)
	—	MCF7	PD-L1 siRNA	Nair et al. (2023)
	Breast cancer/lung metastasis	Autologous breast cancer cells	siS100A4	Zhao et al. (2020)
	Pancreatic cancer	PANC-1	PAK4 siRNA	Xu et al. (2021)
	Ovarian cancer	SKOV3	CRISPR/Cas9-targeting PARP-1	Kim et al. (2017)
	Melanoma	B16BL6	SAV-LA/CpG DNA	Morishita et al. (2016)
Proteins	—	B16BL6	SAV-LA/GALA	Morishita et al. (2017)

Abbreviations: SAV-LA: streptavidin-lactadherin.

### 4.3 Cancer vaccine strategies targeting TDEs

In recent decades, cancer immunotherapy has emerged as a treatment modality that manages and eliminates tumors by modulating the immune system to reinvigorate the anti-cancer immune response, particularly in the realms of CAR-T cell therapy and immune checkpoint blockade (Desai et al., 2024). However, many cancer patients experience significant cytotoxic side effects when undergoing various immunotherapies. These therapies have the potential to trigger widespread immune dysregulation and overstimulation of immune cells, causing a surge in cytokine production that might trigger a cytokine storm (Fajgenbaum David and June Carl, 2020; Shah et al., 2023). Consequently, this cascade of events could lead to substantial harm to tissues and blood vessels, ultimately resulting in the failure of multiple organs. Enhancing the control of the immune response and optimizing therapeutic efficacy can significantly improve the treatment outcomes for individuals with cancer. Therapeutic cancer vaccines, an evolving form of immunotherapy, have the potential to establish long-lasting anti-cancer memory and minimize non-specific or harmful reactions by introducing a variety of tumor antigens (Zhao et al., 2023). This approach ultimately strengthens the immune system’s ability to target and destroy tumor cells. Effective anticancer immunity hinges on the optimal interplay between immune and non-immune elements within the tumor microenvironment. NK cells, neutrophils, and macrophages from the innate immune system play a crucial role in promptly recognizing and attacking tumor cells. Antigen-presenting cells (APCs), such as dendritic cells (DCs), capture and process tumor antigens, presenting them on MHC class II or MHC class I molecules. Subsequently, T cells identify specific antigens on the surface of tumor cells through the T cell receptor (TCR), which binds to peptides displayed by MHC molecules. Once identified, CD8^+^ T cells can directly eliminate tumor cells, while CD4^+^ T cells boost the immune response by releasing cytokines (Luckashenak et al., 2008; Borst et al., 2018; Zhang and Zhang, 2020; Wolf et al., 2023).

Therapeutic cancer vaccines activate the patient’s adaptive immune system to target particular tumor antigens, thereby managing tumor growth, prompting the regression of existing tumors, and eliminating any residual disease present. Successful therapeutic vaccination against tumors relies on several key principles. These include the efficient delivery of substantial amounts of top-notch antigens to DCs, maximizing DC activation, eliciting strong and persistent responses from CD4^+^ T helper cells and cytotoxic T lymphocytes (CTLs), infiltrating the TME, and guaranteeing long-lasting response durability and maintenance (Melief et al., 2002; Aarntzen et al., 2013). DCs are widely acknowledged as the most powerful APCs within the immune system (Palucka and Banchereau, 2012). The crucial contribution of DC vaccines to anti-tumor immunotherapy has garnered growing attention from researchers. Nevertheless, the clinical application of DC vaccines has been somewhat restricted due to the costly, laborious preparation process and quality control, along with factors like the short shelf life and immunosuppressive molecules of patient-derived DCs. DC-derived exosomes (DDEs) are a type of biological nanomedicine that have the potential to replace DC vaccines in stimulating immune responses (Nikfarjam et al., 2020; Zhu H. et al., 2022). The DDEs vaccine, characterized as a non-cellular vaccine, is distinguished by its precisely defined composition, stable structure, excellent safety record, and remarkable capacity for long-term cryopreservation. It effectively mitigates various limitations associated with DC vaccines, positioning itself as the predominant choice among exosome vaccines (Lyu et al., 2024). Nonetheless, the capacity of DDEs vaccines to elicit tumor antigen-specific T cell responses is constrained. Typically, antigen loading or heightened stimulation is necessary to augment T cell activation by DDEs.

TDEs contain various antigens as well as MHC class I and II molecules possessed by the original cancer cells (Wolfers et al., 2001; Andre et al., 2002). Thus, TDEs could modulate the immune system through neoantigen presentation and could be exploited as a cancer vaccine. Rao et al. demonstrated that cancer cell derived exosomes could effectively deliver various tumor antigens to DCs, and then enhanced DCs maturation and antigen presentation, exhibiting better cancer vaccination effects than tumor cell lysates (Rao et al., 2016). Upon taken up by DCs, antigens in TDEs are processed into peptides within the MHC groove, priming naivë T-cells to induce anti-cancer immune responses (Hammerich et al., 2015). Wolfers et al. revealed TDEs contained shared tumor antigens could the activate CTLs, showing antitumor effects on syngeneic and allogeneic tumor challenges (Wolfers et al., 2001). However, TDEs alone application has shown limited immunogenicity and may lead to immune cell suppression and the escapes of immune surveillance due to their inherent cargo promoting tumor growth and metastasis. Therefore, it is essential to increase the immunogenicity of TDEs by modifying their cargo. Utilization of TDEs as endogenous tumor antigens as well as delivery carrier for immunomodulator is attractive to improve cancer immunotherapy. Increasing the expression of natural adjuvant proteins or cytokines in TDEs enhance antitumor immunogenicity effectively. Yoshinobu et al. demonstrated that cytosine-phosphate-guanine (CpG) DNA modification of TDEs exerted stronger antitumor effects than simple co-administration of exosomes and CpG DNA in tumor-bearing mice by efficiently and simultaneously delivering of tumor antigens together with adjuvant to APCs (Morishita et al., 2016). As a potent adjuvant in polarizing T helper type 1 (Th1) cells, HSP70-enriched TDEs produced by heat-stressed tumor cells elicit stronger antitumor immunity than TDEs derived from untreated cancer cells (Dai et al., 2005). Zhang et al. revealed TDEs modified with interleukin-12 (IL-12), a pro-inflammatory cytokine promoting cancer immunotherapy, significantly enhanced T-cell proliferation and subsequent IFN-γ production, efficiently inducing antigen-specific CTLs through the FasL/Fas signaling pathway (Zhang et al., 2010). Overexpressing tumor-specific antigens is another approach to boost immunogenicity. Cho et al. reported that Mucin 1 (MUC1)-expressing on the surface of TDEs exhibited improved activation of dendritic cells and suppressed tumor growth in a dose-dependent manner (Cho et al., 2005) (Table 3).

**TABLE 3:** Summary of research using TDEs as cancer vaccines.

Cancer type	TDE source	Cargo	Evaluation	References
Renal cell carcinoma	RC-2	IL-12	Enhanced T-cell proliferation and IFN-γ production	Zhang et al. (2010)
Colon carcinoma	CT26/TA3HA	hMUC1	Stimulation of immune cells, suppression of tumor growth	Cho et al. (2005)
Pancreatic	Pancreatic cells	NA	Induction of apoptosis in cancer cells via notch signaling	Ristorcelli et al. (2009)
Leukemia	L1210	NA	Inhibition of tumor growth CTL-induced lysis of cancer cells	Bu et al. (2006)
Lymphoma/Leukemia colon	A20/CT-26	HSP	Heat-shocked TDE more efficacious than TEX alone immune response	Chen et al. (2006)
Lymphoma	E.G7-OVA cells	staphylococcal enterotoxin A	Inhibition of tumor growth, increased antigen-specific cytokine secretion by T cells	Xiu et al. (2007)
Colon/Melanoma	CT26-MUC1	HSP70	Upregulation of Th1-mediated tumor response	Cho et al. (2009)
Prostate cancer	RM-1	SA-IFN-γ	Increased the number of M1 macrophages, downregulated the expression of VEGF 2 and attenuated metastasis	Shi et al. (2020)
Melanoma	B16BL6	SAV-LA/CpG DNA	exhibited stronger *in vivo* antitumor effects in Stronger *in vivo* antitumor effects in B16BL6 tumor-bearing mice than simple co-administration of exosomes and CpG DNA	Morishita et al. (2016)
Melanoma	B16F10	Trehalose	More robust antigen-specific immune responses, a stronger capability to inhibit tumor growth	Mu et al. (2024)
Lung tumor	A549/LLC	NA	More robust CTL response, downregulated Tregs *in vitro*, suppressed the tumor growth and prolonged survival rate *in vivo*	Wang et al. (2020a)
Pancreatic ductal adenocarcinoma	PANC-02	MART-1/CCL22 siRNA	Delaying tumor growth	Zhou et al. (2022)

Abbreviations: CTL, tumor-specific cytotoxic T lymphocyte.

Despite the promising results in harnessing TDEs as vaccines, TDEs, are actually double-edged swords as the therapeutic agents. Several aspects still need to be considered before exploring their efficacy in clinical trials, such as the immune cell suppression, the escapes of immune surveillance and the formation of pre-metastatic niche. Therefore, engineered TDEs with appropriate modification provide the cancer researchers in this community with the latest ideas on deliver antitumor drugs to tumor sites efficiently and precisely with fewer treatment-related adverse effects.

### 4.4 Engineered TDEs for other therapy

Due to their spatial and temporal targeting ability, engineered exosomes are attracting increasing attention in cancer therapy fields. Engineered exosomes possess additional synthetic properties that go beyond natural exosomes’ functional contents, ligands, and receptors (García-Manrique et al., 2018). Efficiently engineered TDEs enhance the exocytosis process originating from cells, or boost their natural anticancer properties via external molecular modification, including biological modification, immunological modification, physical modification and chemical modification. Specific targeting peptides, aptamers and antibodies are the primary modification techniques utilized to engineer exosomes, equipping natural exosomes with spatial targeting capabilities (Ishiguro et al., 2020; Fan et al., 2022; Huang X. et al., 2022). By implementing engineering targeting modifications, a more precise targeting of cells or organs can be attained. By targeting the tumor microenvironment factors like acidity and hypoxia through chemical modifications, the release of drugs can be controlled effectively (Kim et al., 2018). In addition, internal agents and external physical interventions, including magnetic fields, laser irradiation, and ultrasound, play a crucial role in achieving precise drug delivery to tumors both spatially and temporally (Zhang M. et al., 2023). And engineering modification technology can be utilized to extract endogenous cargos from TDEs and eliminate oncogenic cargos, thereby significantly enhancing the biosafety of engineered exosomes (Chavda et al., 2023). Thus, TDEs demonstrated superior drug loading capacity, enhanced targeting ability, treatment efficacy, and low cytotoxicity, indicating a promising potential for engineered TDEs as an advanced drug delivery system for cancer treatment (Zhang W. et al., 2023).

Based on this, engineered TDEs can integrate multiple strategies to enhance the conventional and novel therapy ability, such as radiotherapy, photodynamic therapy (PDT), photothermal therapy (PTT), sonodynamic therapy (SDT). For instance, manganese carbonyl (MnCO) loaded nano-TDEs showed admirable performance in active tumor-targeting, mitochondria damage and radio-sensitization therapy both *in vitro* and *in vivo* via facilitating robust CO evolution and consequent ROS generation in response to X-ray irradiation (Zhu et al., 2021). TDEs loaded with the sodium iodide symporter (NIS) protein efficiently deliver the payload to recipient cancer cells to facilitate radioiodine uptake, enhancing the antitumor effects of ^131^I radiotherapy (Son et al., 2019).

In addition to radiotherapy, external laser irradiation equally achieves high therapeutic efficiency, including PDT and PTT. PDT is a non/minimally invasive cancer treatment approach, exploiting the photosensitizer (PS)-generated ROS tokill cancer cells (Xie et al., 2021). Hyuncheol Kim et al. reported tumor-derived Ce6-R-Exo could function as a novel strategy that enables photoacoustic imaging-guided photodynamic and immune-combination therapy for the treatment of cancer (Jang et al., 2021). Chlorin e6 PS loaded TDEs allowed for targeting of tumor cells, and could be visualized by photoacoustic imaging and efficiently generated ROS intercellular to kill the cancer cells under laser irradiation. Apart from traditional PSs, aggregation-induced emission luminogens (AIEgens) have been developed as one of ideal PS due to their excellent photostability, biocompatibility and potentially low biotoxicity (Hu et al., 2018; Sun et al., 2021). Ben et al. developed TDEs for co-delivering AIEgens and proton pump inhibitors (PPI) for tumor combination therapy, by which PPI inhibited cell glutamine metabolism, suppressed tumor cell GSH and ATP production, and ultimately improved the effect of type-I PDT (Zhu D. et al., 2022).

PTT exploits hyperthermia, generated by photothermal agents (PTAs), upon NIR laser radiation, to ablate tumor cells and liberate drug controllably (Zhang et al., 2013; Timko et al., 2014). PTAs loaded into the engineered TDEs could achieve excellent photothermal therapeutic effects under laser irradiation. ICG is one of the common classical PTAs, which could trigger apoptosis in tumor under laser irradiation (Li et al., 2018). However, due to the hydrophobic characteristic of ICG, it is necessary to encapsulate ICG into a drug delivery platform to improve the solubility of ICG (Porcu et al., 2016). Jin et al. developed a TDE-camouflaged porous silicon nanoparticles (E-MSNs) as a drug delivery system for co-loading ICG and DOX (ID@E-MSNs), achieving the synergistic effects of chemotherapy and photothermal therapy against breast cancer (Tian et al., 2020). Black phosphorus quantum dots (BPQDs) showed wide absorption in entire visible light region, making it have a high photothermal conversion efficiency and possess NIR photothermal properties for phototherapy. Due to instability of organic nanomaterials, ultra-small BPQDs with a size of several nanometers attracted much attention in the research of disease diagnosis and treatment (Liu et al., 2021b). BIU-87-derived exosomes packaged with BPQDs exhibit good targeting ability, hence impressive PTT efficiency evidenced by highly efficient bladder cancer ablation *in vivo*. However, therapeutic efficiency of PTT is usually impaired by the limited penetration depth of photothermal agents (PTAs) active in the NIR-I biowindow (750–1,000 nm) and the thermoresistance caused by heat shock protein (HSP) (Zhu et al., 2016; Zou et al., 2016; Zhao et al., 2018). Alternatively, the NIR-II biowindow (1,000–1,350 nm) exhibited deeper tissue penetration than that of the NIR-I biowindow (Antaris et al., 2016). Xueji Zhang et al. developed MCF-7 cells-derived engineered exosomes combining the vanadium carbide quantum dots (V_2_C QDs) PTA (Cao et al., 2019). The developed engineered exosomes exhibited excellent endosomal escape ability, and targeting the and entering into the nucleus to realize low-temperature PTT with advanced tumor destruction efficiency. Compared with quantum dots and organic dyes, photostable and bio-excretable AIEgens is of great significance to the clinical translation. The TDE/AIE NP hybrid nanovesicles provide an alternative artificial targeting strategy for improving tumor diagnosis and PTT (Li et al., 2021).

SDT, a kind of ROS-mediated cancer therapy approach, utilizes ultrasound (US) and sonosensitizers to kill tumors (Kennedy, 2005). Unlike ROS-mediated PDT, which is limited by the penetration depth and damage to normal tissue, SDT could treat deep-seated tumors safely with sonosensitizer-loaded TDEs. TDEs loading sinoporphyrin sodium (DVDMS), an excellent porphyrin sensitizer with both potential therapeutic and imaging applications, exhibited high stability and specificity towards the homotypic tumors, along with highly controlled US-responsive drug release, and boosted reactive oxygen species (ROS) generation to augment SDT (Liu et al., 2019). Engineered TDE-based SDT not only possesses impotent antitumor property, but also exerts synergistic effects while combing with other therapies. For example, engineered TDEs (EXO@CAT NVs), combination catalase (Cat) and Acyl-CoA synthetase long-chain family member 4 (ACSL4) overexpressing 4T1 cells-derived exosomes with sonosensitizer tetrakis (4-carboxyphenyl) porphyrin (TCPP), allow synergistic anticancer treatment of both ferroptosis and SDT with improved efficacy under US stimulation (Wu et al., 2025) (Table 4).

**TABLE 4:** Combined therapy with engineered TDEs.

Therapeutic modalities	Cancer type	TDE source	Cargo	References
Radiotherapy	Breast cancer	4T1	MnCO	Zhu et al. (2021)
	—	Huh7	NIS	Son et al. (2019)
	Breast cancer	4T1	CuPy-Au	Chen et al. (2023)
	Breast cancer	4T1	FeS_2_	Huang et al. (2021)
Photodynamic therapy	Melanoma	MIA-PaCa-2	Chlorin e6	Jang et al. (2021)
	Melanoma	A375	Ir(III) complex/Fe(III) ions	Feng et al. (2023)
	Breast cancer	4T1	TPP	Qian et al. (2024)
	Gastric cancer	MGC803	TBP-2/PPI	Zhu et al. (2022a)
	Breast cancer	4T1	DCPy	Zhu et al. (2020)
Photothermal therapy	Breast cancer	4T1	ICG/DOX	Tian et al. (2020)
	Bladder cancer	BIU-87	BPQDs	Liu et al. (2021b)
	Breast cancer	MCF-7	V_2_C QDs/TAT	Cao et al. (2019)
	Colon cancer	CT26	TT3-oCB	Li et al. (2021)
Sonodynamic therapy	Breast cancer/lung metastasis	4T1	DVDMS	Liu et al. (2019)
	Breast cancer	4T1	TCPP	Wu et al. (2025)

Abbreviations: TPP, 5,10,15,20-Tetraphenylporphyrin; DCPy, (E)-4-(2-(7-(diphenylamino)-9-ethyl-9H-carbazol-2-yl) vinyl)-1-methyl-pyridin-1-ium hexafluorophosphate.

Briefly, engineered TDEs with membrane modification possessed specific tumor-homing properties. Additionally, combining TDEs with other nanomaterials could synergistic with the traditional or novel therapeutic effects. Most importantly, the development of bioinspired or biomimetic exosomes has shed light on the clinic-staged exosome-based drug delivery platform. Finally, in order to capture the dynamics of physiological and pathophysiological activities of TDEs, appropriate imaging strategies are necessary for imaging engineered TDEs *in vivo*, such as exosomes labeling strategies, reporter systems, and microscopy techniques. Looking forward, engineered TDE-mediated combination therapy and diagnosis has a promising perspective for clinical translation.

## 5 Conclusion and perspectives

Mounting evidence indicates that TDEs play critical roles in tumor progression, metastasis, and immunosuppression by regulating components in the TME in an autocrine or paracrine manner. The review focused on emphasizing the biogenesis and biological effect of TDEs in cancer progression. Also, TDEs used in diagnostic and therapeutic settings, including liquid biopsy, clearance strategies, therapeutic drugs as delivery tools and cancer vaccines, and engineered modification was also mentioned. However, there are several outstanding issues that still remain despite the major advancements in the field. First, TDEs are highly heterogeneous and can induce complex biological responses. Markers can vary not only between different types of cancer but also among individual patients, making exosomal quality and specificity control difficult. TDE heterogeneity hinders a comprehensive understanding of their biogenesis, contents, biodistribution, and functions. Moreover, TDEs heterogeneity not only leads to potential adverse effects and safety concerns due to clearing both TDEs and non-TDEs, but also attenuates therapeutic efficacy and leads to tumor progression when as delivery tools. The absence of a streamlined strategy to rapidly identify markers for specific cancer patients hampers the progress of TDE-based cancer interventions and poses a risk of potential side effects. And the heterogeneity of TDEs poses a significant challenge in isolating them for various applications. Existing exosome separation techniques, such as ultracentrifugation, size-based filtration, size-exclusion chromatography, polymer precipitation and immunoaffinity capture, fail to fulfill the criteria for high purity, yield, cost-effectiveness, and reduced volume constraints. Consequently, the absence of standardized separation, quantification and analysis hinders the acquisition of TDEs with high purity and homogeneity, thereby impeding the advancement of exosome research. Understanding the heterogeneity of TDEs will allow us to establish criteria for use of optimal TDEs subpopulations for therapeutics and diagnostics. Considerable time and effort and detection sensitivities and specificities are required to increase precisely characterizing subpopulations of TDEs and their respective molecular cargo. Secondly, TDEs are a double-edged sword for inducing anticancer immunity. On one hand, they naturally create an immunosuppressive environment that hinders the function and penetration of antitumor cells, such as CD8^+^ T cells. This promotes tumor cell behavior by transporting immune checkpoint inhibitors like PD-L1 and pro-tumoral cargos within them, resulting in immune cell suppression and evasion of immune surveillance. On the other hand, these exosomes can also serve as vehicles for presenting tumor antigens and costimulatory signals, thereby potentially enhancing T cell activity and anticancer immune responses. The dual functionality of TDEs underscores the intricate nature of these exosomes, capable of both impeding and boosting immune responses. This duality presents challenges as well as opportunities in the development of cancer treatment strategies. Moreover, in order to advance the development of engineered TDEs for clinical applications, it is essential to address quality and safety concerns related to large-scale production to ensure consistency. Various parameters, including cell culture conditions, isolation, characterization, and storage, need to be carefully managed and regulated during the clinical translation process. More effort in selectively controlling the TDE cargo is required to replace the pro-tumorigenic components for cancer vaccine based on TDEs. Finally, TDE biogenesis and secretion is largely dependent on the types and stages of cancer, there is an urgent need to perform a high-content screening platform that mimic the cancer microenvironments in a 3D context or more tools for animal studies to model physiological concentrations of TDEs and their role in cancer pathology. The generation of further sophisticated genetic models will allow dynamic, longitudinal studies of TDEs biology *in vivo*, and can contribute comprehensive knowledge of the biogenesis, biodistribution and cellular uptake of TDEs.

To conclude, TDEs play essential roles in tumor progression. More detailed mechanistic on how specific signaling pathway regulates the biogenesis and secretion of TDEs in malignant cancer cells and how oncogenic-specific packaging in TDEs at various stages occurs is required to specifically inhibit the capability of TDEs in promoting disease progression. More importantly, how to combine engineered exosomes, cancer cell-derived, nanomaterial-encapsulated, and aptamer-coated exosomes with external irradiation for clinical cancer-targeted therapy in the future needs more exploration.
